# Epidermal Lamellar Body Biogenesis: Insight Into the Roles of Golgi and Lysosomes

**DOI:** 10.3389/fcell.2021.701950

**Published:** 2021-08-12

**Authors:** Sarmistha Mahanty, Subba Rao Gangi Setty

**Affiliations:** Department of Microbiology and Cell Biology, Indian Institute of Science, Bengaluru, India

**Keywords:** epidermal lamellar body, lysosome-related organelle, Golgi, lysosome, epidermis

## Abstract

Epidermal lamellar bodies (eLBs) are secretory organelles that carry a wide variety of secretory cargo required for skin homeostasis. eLBs belong to the class of lysosome-related organelles (LROs), which are cell-type-specific organelles that perform diverse functions. The formation of eLBs is thought to be related to that of other LROs, which are formed either through the gradual maturation of Golgi/endosomal precursors or by the conversion of conventional lysosomes. Current evidence suggests that eLB biogenesis presumably initiate from *trans*-Golgi network and receive cargo from endosomes, and also acquire lysosome characteristics during maturation. These multistep biogenesis processes are frequently disrupted in human skin disorders. However, many gaps remain in our understanding of eLB biogenesis and their relationship to skin diseases. Here, we describe our current understanding on eLB biogenesis with a focus on cargo transport to this LRO and highlight key areas where future research is needed.

## Highlights

- Epidermal lamellar bodies (eLBs) belong to LRO family and are required for the skin barrier function.
- Epidermal differentiation alters the morphology and dynamics of intracellular organelles and produces eLBs, majorly observed in the granulosum layer of the skin.
- The biogenesis of eLBs possibly initiate from *trans*-Golgi network and acquire lysosomal characteristics during maturation.
- The formation of functional eLBs requires cargo transport from Golgi and endosomes.

## Introduction

**FIGURE 1:**
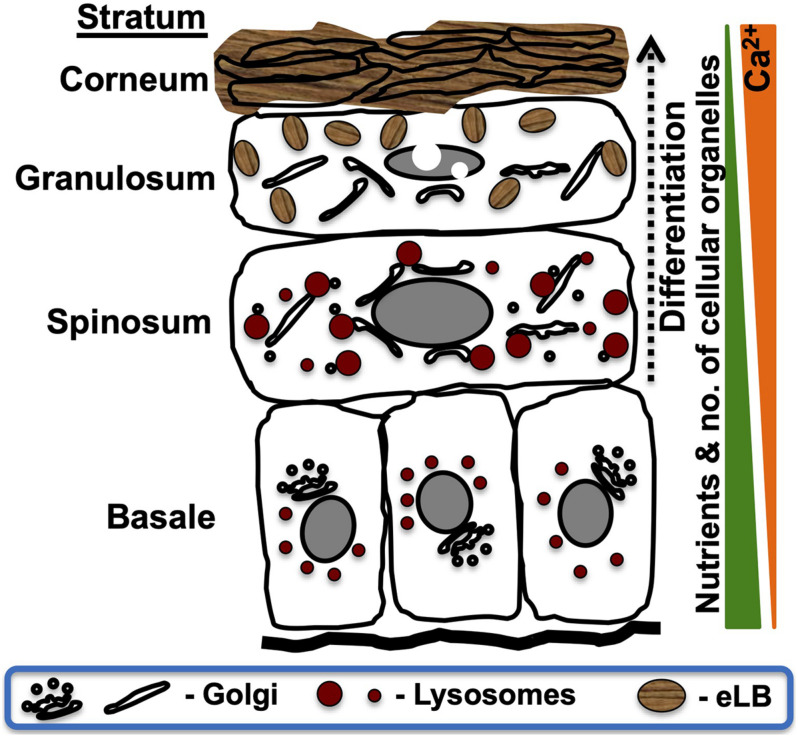
Schematic representation of intracellular organelle dynamics in the epidermal sublayers. The human epidermis is composed of four functionally distinct sublayers formed by variable differentiation states of keratinocytes. Alterations in the extracellular composition and intracellular organelles of keratinocytes are represented according to the established studies (reviewed in Bikle et al., 2012). Stratum basale contains proliferative keratinocytes having all intracellular organelles similar to other cell types. These cells undergo differentiation (dotted arrow) after each cycle of asymmetric cell division and modify the Golgi organization and disperse lysosomes that result in the formation of the spinosum layer (Monteleon et al., 2018; Mahanty et al., 2019; Yamanishi et al., 2019). These cells are further subjected to late-stage differentiation and gradually lose their intracellular organelles while generating a new set of organelles called lamellar bodies (eLBs), which form the granulosum layer. These eLBs undergo exocytosis and generate the lipid sheets/barrier. Lastly, the cells of the granular layer undergo terminal differentiation, which forms SC (reviewed in Bikle et al., 2012). These cells are also called corneocytes (lack intracellular organelles) that remain embedded in lipid sheets produced by eLBs of the granular layer. The sequential changes in intracellular organelle dynamics, including the nucleus, are shown in the sublayers of skin (Akinduro et al., 2016). Extracellular ascending calcium gradient (Ca^2+^) and a descending nutrient gradient exist from the base of the epidermis. Note that the deformation of the nucleus initiates at the spinosum layer and eliminated through nucleophagy in the upper layers of the epidermis.

Intracellular organelles play a crucial role in maintaining skin architecture and homeostasis. Epidermal lamellar bodies (also called lamellar granules, hereinafter referred to as eLBs) are the critical organelles that regulate the formation and maintenance of the skin barrier (reviewed in Feingold, 2007, 2012). Human epidermis consists of four functionally distinct sublayers, formed majorly by keratinocytes, which undergo successive differentiation in response to nutrient stress and an active extracellular calcium gradient. The sublayers of the epidermis, namely, stratum basale, stratum spinosum, stratum granulosum, and stratum corneum, display distinct cellular morphology and organelle organization (Figure 1; Yamanishi et al., 2019; reviewed in Bikle et al., 2012). eLBs are primarily observed in the granulosum layer keratinocytes. These organelles undergo exocytosis to create and maintain the skin barrier in the stratum corneum (SC), which is composed of terminally differentiated keratinocytes (called corneocytes). The SC-associated extracellular matrix is enriched with neutral lipids and antimicrobial peptides (AMPs) that are secreted by eLBs (Figure 1; reviewed in Elias and Choi, 2005; Elias, 2007; Feingold, 2007; Proksch et al., 2008; Feingold, 2012). Thus, eLBs are key organelles for epidermal barrier and antimicrobial properties. Therefore, understanding eLB formation is critical for understanding normal functioning of the skin.

## Epidermal Structure, Homeostasis, and the Skin Barrier

Keratinocytes are the primary cell type of the epidermis, which accounts for approximately 95% of total epidermal cells. Stratum basale, which is the base of the epidermis, contains proliferative keratinocyte stem cells. Overlying the stratum basale is the stratum spinosum, stratum granulosum, and stratum corneum, which is composed of commenced, advanced, and terminally differentiated keratinocytes, respectively (Figure 1; reviewed in Schmitz and Muller, 1991; Bikle et al., 2012). The cells of the SC, called corneocytes, are highly specialized. They lack intracellular organelles and are enclosed by a thick, mechanically stable protein–lipid envelope called the cornified envelope (CE). The major components of the CE are the structural proteins involucrin and loricrin, which are cross-linked by the enzymes sulfhydryl oxidases and transglutaminases (Sun and Green, 1976). In addition, the corneocytes are tightly connected to each other by corneodesmosomes, and the intercellular space is filled with lipid lamellae and AMPs that contribute to the skin barrier and provide antimicrobial protection (discussed in detail below) (reviewed in Ishida-Yamamoto and Iizuka, 1998; Kalinin et al., 2002; Elias and Choi, 2005; Gallo and Hooper, 2012). The skin barrier is periodically renewed by a process called desquamation mediated by the desquamatory proteases (reviewed in Madison, 2003; Proksch et al., 2008). Altogether, the successive differentiation of keratinocytes contributes to the generation of a mechanically stable, hydrophobic, and defensive skin barrier.

Epidermal homeostasis refers to the maintenance of the various sublayers, cornification, and desquamation (reviewed in Proksch et al., 2008; Feingold, 2012). Several studies have shown that the gradient of calcium that spans the epidermis regulates gradual differentiation across epidermal sublayers (Figure 1; Hennings et al., 1989; also reviewed in Bikle et al., 2012). Based on this fact, many *in vitro* studies utilizing human primary keratinocytes use calcium to trigger the differentiation process (Hennings et al., 1989; Tu et al., 2007, 2012; Toufighi et al., 2015; Mahanty et al., 2019; reviewed in Bikle et al., 2012; Elsholz et al., 2014). Note that the epidermal sublayers having eLBs are only observed *in vivo* or in three-dimensional (3D) skin models (Pillai et al., 1988; Boyce and Williams, 1993; Fartasch and Ponec, 1994). Although the differentiated primary keratinocytes do not produce eLBs, they recapitulate many *in vivo* characteristics found *in vivo* and in 3D skin models. For instance, autophagy is reported to be indispensable for both *in vitro* keratinocyte differentiation and epidermal homeostasis *in vivo* (Aymard et al., 2011; Rossiter et al., 2013; Sukseree et al., 2013b; Yoshihara et al., 2015; Mahanty et al., 2019). Additionally, the morphological characteristic of intracellular organelles like dispersed Golgi is observed in calcium-induced differentiated keratinocytes (Mahanty et al., 2019), similar to the reticular Golgi seen in human skin layers (Yamanishi et al., 2019). Hence, *in vitro* models of primary keratinocytes recapitulate many aspects of *in vivo* skin development (reviewed in Bikle et al., 2012) and may be useful for studying the initial stages of eLB biogenesis.

## eLBs Linking Skin Barrier Homeostasis

Epidermal impermeability toward water/electrolytes and the barrier against the pathogenic microorganisms are carried out by the SC (reviewed in Elias and Choi, 2005; Elias, 2007; Feingold, 2007). The hydrophobic barrier of the SC is composed of thick lipid sheets containing ceramides (50%), cholesterol (25%), and free fatty acids (15% having 22–24 linear carbon chains; reviewed in Bouwstra and Ponec, 2006; Wertz, 2006; Elias, 2007; Feingold, 2012). These lipids are secreted as precursors, which are then processed into their respective lipid products by enzymes that are cosecreted by the eLBs (Table 1; reviewed in Madison, 2003; Elias and Choi, 2005). For example, β-glucocerebrosidase and sphingomyelinase convert the glucosylceramide and sphingomyelin, respectively into ceramide, the major constituent lipid of SC (Holleran et al., 1993, 1994; Jensen et al., 1999; Schmuth et al., 2000). Similarly, phospholipase A2 converts phospholipids into free fatty acids and glycerol, and steroid sulfatase hydrolyzes cholesterol sulfate into cholesterol (Mao-Qiang et al., 1995, 1996; Elias et al., 2004). These lipid metabolites contribute to skin health. For instance, glycerol acts as a water-retaining agent, and cholesterol metabolites help in desquamation of the skin barrier (reviewed in Elias, 2007; Feingold, 2007). In addition, the free fatty acids produced by phospholipid digestion also contribute to the antimicrobial barrier by maintaining the acidic pH of 5.5 in the SC (Fluhr et al., 2001, 2004a,b; reviewed in Mauro, 2006). eLBs also secrete AMPs such as cathelicidin LL37 and human β-defensin 2 (Table 1). These AMPs remain bound to the extracellular lipids and form the antimicrobial barrier (Oren et al., 2003; Braff et al., 2005). Finally, eLBs secrete a variety of proteolytic enzymes (such as cathepsins, serine protease enzymes of the kallikrein family) and enzyme inhibitors (such as SPINK5 gene product; Table 1), which control the desquamation of the skin barrier (reviewed in Madison, 2003; Proksch et al., 2008). Thus, eLBs perform a multitude of biological functions and are essential in the formation and maintenance of the skin barrier. In accordance with their important functions, deregulation of the formation and/or secretion of eLBs is associated with defective skin barrier and transepidermal water loss (TEWL), which is linked to several cutaneous diseases, including atopic dermatitis (reviewed in Tagami and Kikuchi, 2006; Feingold, 2007, 2012; Elias and Wakefield, 2014; Menon et al., 2018). Moreover, the eLB abnormalities are directly associated with or proposed to cause several life-threatening diseased conditions such as Harlequin-type ichthyosis and severe ichthyosis in CEDNIK (cerebral dysgenesis, neuropathy, ichthyosis and keratoderma) and MEDNIK (developmental delay, enteropathy, deafness, peripheral neuropathy, ichthyosis, and keratodermia) syndromes (discussed below; reviewed in Ishida-Yamamoto et al., 2018).

**TABLE 1:** List of cargoes specific to eLBs and shared with the lysosomes.

Cargo	Function	Associated cellular process	References
**Cargoes specific to eLBs**			
ATP-binding cassette sub-family A member 12 (ABCA12)	Lipid transporter	Formation of the skin barrier	Akiyama, 2011
Corneodesmosin (CDSN)	Adhesion glycoprotein of corneodesmosome	Formation of corneodesmosome	Jonca et al., 2002
Cathelicidin/LL-37	Antimicrobial peptide	Antimicrobial defense	Braff et al., 2005
β-defensin 2 (BD-2)	Antimicrobial peptide	Antimicrobial defense	Oren et al., 2003
Kallikrein-related peptidases (KLK): 5, 7, 14	Serine proteases	Desquamation	Borgono et al., 2007
Serine protease inhibitor Kazal-type 5 (SPINK5)	Serine protease inhibitor	Regulation of desquamation	Elias and Wakefield, 2014
**Molecules associated with eLBs**			
Rab11A	Rab GTPase	eLB maturation or secretion?	Ishida-Yamamoto et al., 2007; Reynier et al., 2016
CLIP-170/restin	Peripherally associated to the limiting membrane	eLB biogenesis or secretion?	Raymond et al., 2008
**Cargoes common to eLBs and lysosomes**			
Glucosylceramide Sphingomyelin Phospholipid Cholesterol	Lipid precursors	Formation of stratum corneum and maintenance of skin barrier	Reviewed in Elias et al. (2006); Feingold (2012)
β-glucocerebrosidase Sphingomyelinase Phospholipase A2 Steroid sulfatase	Lipid hydrolases	Formation of stratum corneum and maintenance of skin barrier	Reviewed in Elias et al. (2006); Feingold (2012)
Cathepsin D	Protease	Desquamation	Raymond et al., 2008
LAMP-1 LAMP-2 LIMP-2	Structural membrane proteins/receptor	eLB biogenesis?	Raymond et al., 2008
Rab7 Arl8b	GTPases peripherally associated to the limiting membrane	eLB biogenesis?	Raymond et al., 2008

**“?” the cellular processes associated with these proteins require investigation.**

## eLBs Are Specialized Lysosome-Related Organelles

LROs are defined as discrete membrane-bound organelles that have an acidic pH and contain a variety of lipid and protein hydrolases as similar to lysosomes (reviewed in Schmitz and Muller, 1991; Elias et al., 2006; Feingold, 2007). Based on these characteristics, eLBs are classified as LROs (Schmitz and Muller, 1991) or ELROs (endo-LROs) (Delevoye et al., 2019). In addition to the lysosomal enzymes, eLBs also carry specific cargoes that contribute to the skin barrier formation. Importantly, these organelles encode significantly distinct proteome in comparison with other LROs (Ridsdale et al., 2011). In addition, although it is unknown whether eLBs are affected in multiorganellar disorders like Chediak–Higashi and Hermansky–Pudlak syndromes (reviewed in Boissy and Nordlund, 1997; Spritz, 1999), the mouse models and human patients of these diseases do not exhibit notable epidermal changes other than pigmentation or coat color defects (reviewed in Wei, 2006; Huizing et al., 2008). Nonetheless, it will be worth analyzing epidermal architecture, including the functional status of eLBs, in these disease models.

**FIGURE 2:**
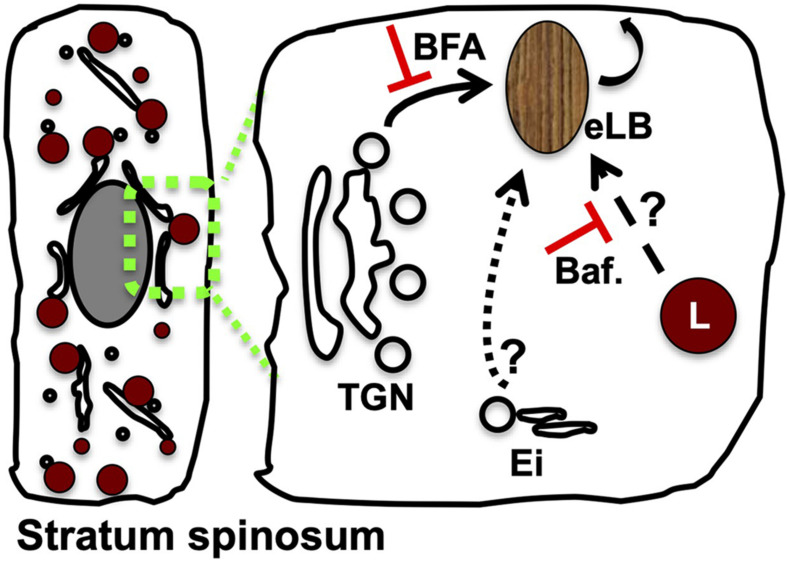
Modulation of Golgi or lysosome function alters the epidermal LB biogenesis and homeostasis. A set of TGN-derived vesicles possibly acts as the precursors of eLB in the differentiated keratinocytes of the spinosum layer, which matures into functional LBs post sequential maturation by receiving the cargo/membranes from the endolysosomal system (indicated as arrows). Several studies support this model: (a) inhibition of Golgi function with brefeldin A (BFA) blocks (shown as red color T) the production of epidermal lysosomes (L) (Mahanty et al., 2019) and LBs (Madison and Howard, 1996) and also inhibits keratinocyte differentiation (Mahanty et al., 2019); (b) inhibition of lysosome function by using bafilomycin (Baf.) causes defective epidermal architecture (shown as red color T) (Monteleon et al., 2018) and also inhibits the keratinocyte differentiation (Mahanty et al., 2019); and (c) silencing of Rab11A or knockout mouse of VPS33B or VIPAR displays defective eLB biogenesis, and barrier function suggests a role for Ei (consists of early/recycling endosomes) during their biogenesis (Reynier et al., 2016; Rogerson and Gissen, 2018). The drawn hypothetical model is based on the stepwise cargo transport to maturing TGN-derived precursor organelles, which generate functional eLBs. Differentiated keratinocyte of the stratum spinosum layer was shown on the left. The possible cargo trafficking routes to eLBs are indicated in the enhanced portion on the right side.

Several studies have suggested that eLBs originate from the *trans*-Golgi network (TGN); however, the exact mechanism of their biogenesis remains unknown (Figure 2; Madison and Howard, 1996; Madison et al., 1998; Tarutani et al., 2012). Technically, eLBs are rare and difficult to produce in 2D cellular models (Pillai et al., 1988), which is a major hurdle to study their biogenesis. *In vivo* eLBs are abundant in the granular layer, where other intracellular organelles are eliminated through elevated autophagy (Yoshihara et al., 2015; Akinduro et al., 2016; Monteleon et al., 2018; Mahanty et al., 2019; reviewed in Sukseree et al., 2013a; Eckhart et al., 2019). This suggests a strong correlation between production of eLBs and the destruction of other intracellular organelles within the epidermal sublayers. In addition, in both cellular and tissue models of the skin, a marked change in number, size, and distribution of intracellular organelles, especially the lysosomes and Golgi, were observed (Mahanty et al., 2019; Yamanishi et al., 2019; reviewed in Schmitz and Muller, 1991). These changes may contribute to the eventual formation of eLBs. Thus, studying the sequential alterations of Golgi and lysosomes in the epidermal layers may provide potential clues toward the eLB biogenesis.

## Ultrastructural Analysis of eLB Morphology

LROs are diverse organelles that perform distinct functions in different cell types. LROs mature via different pathways, often emerging from endosomal precursors or TGN. During this gradual maturation process, these intermediates acquire the unique cargo, lysosomal proteins, and acidic pH (reviewed in Raposo et al., 2007; Marks et al., 2013; Bowman et al., 2019; Delevoye et al., 2019). Several studies have suggested that eLBs emerge from TGN, based on the appearance of eLBs as oval, elongated/oddly shaped granules under electron microscopy (EM). These structures are more similar to the EM cross sections of TGN tubules or buds (reviewed in Schmitz and Muller, 1991; Madison et al., 1998; Norlen, 2001a). In support of this hypothesis, the pharmacological inhibition or genetic alteration of Golgi disrupts eLB formation (Madison and Howard, 1996; Tarutani et al., 2012). However, further studies are required to connect the role of Golgi directly to the formation of eLBs (see below).

**FIGURE 3:**
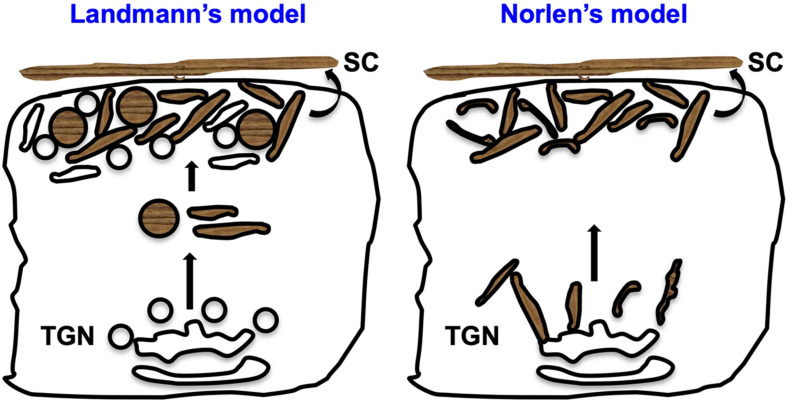
Proposed models for lipid matrix formation at the SC. Landmann’s model: In the upper spinosum and granular layer keratinocytes, eLBs are bud off from the TGN as discrete vesicles/tubules. The cargo in these vesicles is packaged in the form of disks that avoid the enzyme activity during their transport. These vesicles fuse with the plasma membrane of the granular cells and release the disks between the SG-SC junction, which merge together and generate the lipid sheets of the corneum layer (Landmann, 1986; Norlen, 2001a). Norlen’s model: eLBs are the continuous tubulo-reticular network from TGN to the plasma membrane and multilamellar lipid matrix at the SG-SC junction. Further, these organelles secrete lipids through the intersection-free unfoldings at the plasma membrane invaginations of the granular cells (Norlen, 2001a). In both these models, keratinocytes of the SG secrete eLBs (either in the form of vesicles or tubules), which form the SC layer.

Prior studies have described two different models for the formation of lipid matrix at the SC, mostly based on eLB morphology and their method of secretion (Figure 3). (1) Landmann’s model: eLBs are formed as discrete vesicles via membrane budding following fission from the TGN, and these vesicles enclose cargo in separate disks possibly to avoid enzyme activity during their transport. These vesicles undergo exocytosis by fusion with the plasma membrane of granulosum layer cells, and the disks enclosing lipid cargo are released into the stratum granulosum (SG)–SC junction. Following secretion, these discs merge with each other and generate continuous and uninterrupted lipid sheets of SC post digestion by the cosecreted enzymes released from eLBs (Landmann, 1986; Elias et al., 1998). (2) Norlen’s model: eLBs form a continuous tubulo-reticular network of TGN that is connected to the plasma membrane of granular layer cells and the multilamellar lipid matrix. These organelles secrete lipids via a single-step unfolding of the plasma membrane invaginations at the SG-SC junction (Norlen, 2001b; den Hollander et al., 2016). However, the model does not describe the mechanism of cargo packaging or transport. Nevertheless, Norlen’s model is biologically favorable over Landmann’s model because it requires less energy and time, as it involves fewer fission/fusion steps and is able to maintain the membrane continuity (reviewed in Norlen, 2001a). Interestingly, a recent *in vivo* study showed eLB’s morphology change from ovoid to reticular in the upper granular layers in skin samples by using reconstructed focused ion beam scanning EM (Yamanishi et al., 2019). This indicates that eLB morphology may differ in different epidermal layers. Altogether, these studies present several opinions on eLB morphology and their secretion process.

## Cargo Sorting to eLBs With a Perspective to Other LROs

The entry of lipids into the eLBs is an important and unique aspect of their biology. Glucosylceramide is the major lipid content of eLBs, along with phospholipids and cholesterol (Table 1). The ABC transporter ABCA12 is known to transport glucosylceramide into maturing eLBs, and mutations in ABCA12 result in pathologies like severe lamellar ichthyosis, Harlequin-type ichthyosis, and other diseases, indicating the importance of lipid import during the eLB formation (reviewed in Akiyama, 2011, 2014; Scott et al., 2013). In contrast, the mechanism of phospholipid and cholesterol transport to eLBs has not yet been demonstrated. However, ABCG1 has been implicated in cholesterol transport since the null mice displayed abnormal eLB content and secretion (Jiang et al., 2010). Interestingly, the transport of structural proteins and enzymes to maturing eLBs occurs concomitantly or post incorporation of lipid precursors (reviewed in Feingold, 2007). Further, the inhibition of lipid synthesis leads to a block in protein/enzyme transport to eLBs, and the exogenous supply of lipids restores the biogenesis process, suggesting the importance of prior lipid transport during eLB biogenesis (Rassner et al., 1999). Hence, the preexistence of glucosylceramide containing eLB precursors and their sequential maturation may form a requisite for the generation of functional eLBs.

It remains unclear how protein cargo is sorted into eLBs. Ishida-Yamamoto et al. (2004) have found different eLB cargoes form distinct aggregates in the branched tubular structures of the TGN, by monitoring different eLB cargoes using immunoelectron microscopy. The process of cargo aggregation is observed in the formation of LROs such as Weibel–Palade bodies (WPBs) in endothelial cells and lamellar bodies in type II lung epithelial cells (hereinafter referred to as lung lamellar bodies or lLBs) (reviewed in Dell’Angelica et al., 2000; Raposo et al., 2007; Delevoye et al., 2019). For WPBs, the major cargo protein von Willebrand factor (VWF) accumulates in tubules at the TGN. This accumulation is critical for the biogenesis of WPBs because disruption of VWF tubules leads to altered WPB morphology and impaired function (Wagner et al., 1991; Michaux et al., 2006; reviewed in Michaux and Cutler, 2004). In the case of lLBs, the cargo surfactant protein B (SP-B) accumulates during their biogenesis from the endosomal network (Weaver et al., 2002). In line, the knockout mice of SP-B displayed morphologically and functionally abnormal lLBs, suggesting the existence of the major cargo-dependent biogenesis and maturation mechanism (Stahlman et al., 2000). Taken together, cargo aggregation and prior lipid transport to the maturing organelles are the mechanisms opted by certain LROs of both Golgi and endosomal origin.

A defining characteristic of all LROs is the presence of lysosomal membrane proteins and hydrolases (reviewed in Dell’Angelica et al., 2000). It is unclear how eLBs receive these cargoes. Classical lysosomes receive acid hydrolases from the TGN (directly or via plasma membrane) by the action of receptors such as mannose 6-phosphate receptor, LIMP-2, etc. (reviewed in Kornfeld and Mellman, 1989; Pfeffer, 1991; Luzio et al., 2003; Saftig and Klumperman, 2009). On the other hand, lysosomal membrane proteins such as LAMP-1 and LAMP-2 are transported from the TGN to lysosomes in clathrin-coated AP-1 vesicles (Luzio et al., 2014). In addition, a direct route from the TGN to late endosomes is described and mediated by a special class of uncoated vesicles (Pols et al., 2013). However, it is unknown whether eLBs receive the lysosomal cargo by using these mechanisms.

An alternative mechanism for directing the lysosomal proteins to LROs is via the modification of existing conventional lysosomes. In this process, no special sorting machinery is required for their biogenesis. Therefore, it is essential to understand the lysosome cross talk with eLBs to understand the cargo transport mechanisms to eLBs. It should be noted that the appearance of eLBs correlates with the expression of glucosylceramide synthase (GCS) in the Golgi. However, no such correlation was observed with the expression of lysosomal hydrolases, including β-glucocerebrosidase (Madison et al., 1998). These results are furthering the concept of TGN origin of eLBs and acquiring the lysosomal cargo through the endosomal route. Thus, understanding the cross talk between eLBs, Golgi, and lysosomes in parallel will possibly open up the path to eLB biogenesis. Hence, we have discussed current understanding of the roles of Golgi and lysosomes with respect to eLB biogenesis and skin barrier homeostasis.

## Key Roles of Golgi Toward eLBs Biogenesis and Epidermal Homeostasis

**FIGURE 4:**
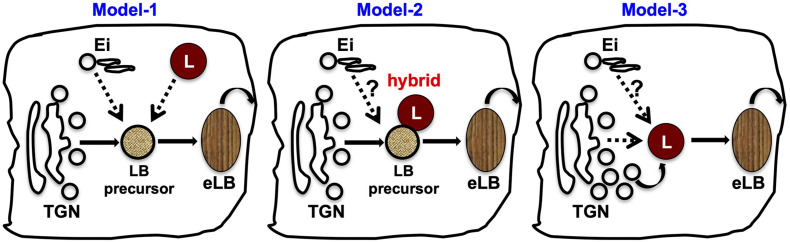
Current models for the biogenesis of epidermal LBs. Based on the existing cargo/membrane transport routes to eLBs, we propose three variant mechanisms for the biogenesis of eLBs in the upper spinosum and granulosum layer keratinocytes. Model-1: LB precursors are derived from the TGN, which mature into functional eLBs upon receiving the cargo/membranes from early/recycling endosomes (Ei) and epidermal lysosomes (L) in a sequential manner. Model-2: TGN-derived LB precursors fuse with epidermal lysosomes and form hybrid organelles, which then mature into eLBs. The cargo transport from the early/recycling endosomes (Ei) to this hybrid organelles is not clear in this model. Model-3: The epidermal lysosomes of the spinosum layer mature into eLBs upon constant fusion of TGN-derived vesicles. As similar to model-2, the input from early/recycling endosomes (Ei) is not clear in this pathway. In all the three models, the matured eLBs fuse with the plasma membrane to deliver its content for the formation of lipid sheets at the SC.

The TGN is the primary organelle for the biogenesis of several LROs, and it facilitates cargo transport during their maturation. Similarly, several lines of evidence support a critical role for the TGN in eLB biogenesis. First, disruption of Golgi by small-molecule inhibitor brefeldin A inhibits eLB biogenesis (Madison and Howard, 1996; Rassner et al., 1999); likewise, the knockout mouse of Golgi pH regulator (*GPR89*) displayed defective eLB formation and skin barrier function (Tarutani et al., 2012). In addition, the synthesis of the major eLB cargo glucosylceramide takes place in the Golgi by the enzyme GCS. The enhanced GCS expression corresponds to the increased eLB appearance (Madison et al., 1998). Finally, Golgi morphology is dramatically altered in the granulosum layer enriched with eLBs (Yamanishi et al., 2019). Based on these findings, we propose a model for eLB biogenesis (Figure 4, Model-1 and –2, discussed below), wherein glucosylceramide containing TGN-derived vesicles possibly act as precursors of eLBs followed by their sequential maturation upon receiving endolysosomal input or fusion with the lysosomes.

In addition to its role in eLB biogenesis, the Golgi also controls the keratinocyte differentiation and epidermal homeostasis. Accordingly, the depletion of Golgi ion pumps and channels that alter Golgi’s function results in defective epidermal homeostasis (Sudbrak et al., 2000; Behne et al., 2003; reviewed in Wuytack et al., 2003; Shull et al., 2011). Furthermore, the altered Golgi morphology may fulfill the secretory needs of differentiated keratinocytes and the epidermis (Elias et al., 1998; Yamanishi et al., 2019). Taken together, accumulating recent evidence supports a role for Golgi in the generation of eLBs and epidermal homeostasis.

## The Role of Endosomal Trafficking Machinery in eLB Biogenesis

Membrane traffic likely plays several critical roles in eLB formation. Potential biosynthetic mechanisms include sequential maturation or direct fusion of Golgi-derived eLB precursors with the endosomal vesicles (Figure 4). Indeed, mutations in several genes important for trafficking cause skin disorders, some of which have clear effects on eLB biogenesis, highlighting the importance of cargo traffic during eLB biogenesis. Severe defects in eLB formation and epidermal differentiation occur in response to mutations in the SNARE SNAP29, resulting in severe ichthyosis in CEDNIK syndrome (Sprecher et al., 2005; Fuchs-Telem et al., 2011; Schiller et al., 2016). Similarly, a role for clathrin adaptor AP-1 in eLB maturation or biogenesis is suggested by the similarities of disease phenotypes between CEDNIK and MEDNIK syndrome, which is caused by a mutation in the sigma subunit of AP-1 (AP1S1) (Montpetit et al., 2008; reviewed in Martinelli and Dionisi-Vici, 2014). However, the effect of AP-1 mutations on eLB formation or function has not been examined directly. Collectively, these studies strongly indicate the role of endosomal sorting and fusion machinery in the eLB maturation. In accordance with this model, mutations in VPS33B cause a severe ichthyosis condition as reported in ARC (arthrogryposis, renal dysfunction, and cholestasis) syndrome (Hershkovitz et al., 2008). VPS33B and its interactor VIPAR or VIPAS39 constitute the CHEVI (class C Homologs in Endosome-Vesicle Interaction) complex, which functions in the early endosomal pathway and is involved in the biogenesis of alpha granules, integrin recycling, and cell polarity (Cullinane et al., 2010; Xiang et al., 2015; Dai et al., 2016; reviewed in Rogerson and Gissen, 2016). Knockout mouse models of VPS33B and VIPAR display skin phenotype similar to ARC patients and display phenotypes associated with defective keratinocyte differentiation and cornified envelop formation. Importantly, the VPS33B- and VIPAR-deficient mice show abnormal eLB formation, which is associated with decreased lipid deposition in the SC sublayer following defective SC formation (Rogerson and Gissen, 2018).

Moreover, the CHEVI complex interacts with GTPase Rab11A, a component of the recycling endosomes (Cullinane et al., 2010), which regulates the trafficking of eLB cargo to the cell surface (Ishida-Yamamoto et al., 2007). Consistent with a role of Rab11A in eLB biogenesis, silencing of Rab11A in *in vitro* constituted human epidermis causes defective skin barrier due to altered eLB biogenesis or its secretion, which reduces the lipid deposition in the SC (Reynier et al., 2016). These results suggest that the Rab11A and CHEVI complex may function together to regulate the maturation of eLBs. Although the exact mechanism is unclear, potentially these molecules may facilitate the fusion of a nascent eLB with the early endocytic organelles and/or regulate the fusion of eLBs with the plasma membrane. Consistent with this latter possibility, a recent study showed that Rab11 functions in a cascade with Rab3A to regulate the lysosomal exocytosis by interacting with Sec15, a subunit of the exocyst complex and GRAB, a GEF for Rab3A (Escrevente et al., 2021). The role of Rab27A in regulating the secretion of several LROs has been established in cytotoxic T lymphocytes, neutrophils, endothelial cells, and melanocytes (reviewed in Luzio et al., 2014). Here, we speculate that Rab27A may play a role in concert with the Rab11A-CHEVI complex during the secretion of eLBs. Lastly, the intracellular trafficking of eLBs has been shown to be regulated by CLIP-170/restin along with Cdc42 and/or Rab7, suggesting a role for these molecules in the biogenesis of eLBs (Raymond et al., 2008). Overall, these studies suggest that the eLBs receive the cargo/membranes of early/recycling endosomes [referred to here as endosomal input (Ei)] at a certain point of time during their biogenesis, highlighting the crucial role of Ei in the biogenesis of eLBs (Figure 4).

## Key Roles of Lysosomes Toward eLB Function

eLBs are classified as LROs. Hence, an understanding of the functional cross talk of eLBs with the lysosomes may provide potential clue toward eLB biogenesis. Established information suggests the functional cross talk between classical lysosomes and epidermal homeostasis: (a) genetic or pharmacological inhibition of lysosome function leads to defective epidermis formation and barrier function in 3D organoid cultures (Monteleon et al., 2018; Bochenska et al., 2019), and (b) lysosomal dysfunction is associated with the diseased conditions such as psoriasis and atopic dermatitis (Klapan et al., 2021). Together, these studies suggest that the functional lysosomes are required for epidermal homeostasis.

On the other hand, lysosomes play a crucial role in the formation of the skin barrier by supporting high flux of macroautophagy, which is required for the removal of intracellular organelles from upper layers of the epidermis (Rossiter et al., 2013; Yoshihara et al., 2015; Akinduro et al., 2016; Monteleon et al., 2018; Bochenska et al., 2019; reviewed in Sukseree et al., 2013a; Eckhart et al., 2019; Murase et al., 2020). Inline, inhibition of lysosome function results in impaired epidermal homeostasis (Monteleon et al., 2018). Consistent with this, upregulation of the lysosomal network is reported during *in vitro* keratinocyte differentiation (Tanabe et al., 1991; Tobin et al., 2002; Jans et al., 2004; Sarafian et al., 2006; Mahanty et al., 2019), which is implicated in various cellular processes including plasma membrane repair (Reddy et al., 2001; Jans et al., 2004; Appelqvist et al., 2013). Collectively, these studies provide the importance of lysosome function in the maintenance of epidermal homeostasis.

Although adequate EM studies illustrated the structural features of eLBs but not lysosomes, especially in the granulosum layer of the epidermis (Wolff and Schreiner, 1970; Ishida-Yamamoto et al., 2004), these observations possibly suggest the absence of classical lysosomes in the granular layer that remain to be investigated. Based on this, we speculate a model in which the lysosomes of the spinosum layer may convert into eLBs during the progressive keratinocyte differentiation, (or) eLBs are hybrid organelles of endolysosomal origin formed post fusion of Golgi vesicles (Figure 4, Model-3). Aligned with this model, a proteomic analysis by using nano liquid chromatography with tandem mass spectrometry (LC-MS-MS) has detected several lysosomal proteins such as hydrolases (β-glucocerebrosidase) and lysosomal structural proteins (LAMP-1, LAMP-2, and LIMP-2) in the isolated fraction of eLBs (Table 1; Raymond et al., 2008). Nevertheless, these observations pose several open questions that remain to be investigated in the future: (1) How is the high autophagy flux supported in the absence of lysosomes in the granular layer? and (2) Do the lysosomes coexist with eLBs in upper skin layers?

## Future Prospective

A long-awaited question in the field of skin biology is the mechanism of epidermal LB biogenesis, which includes membrane origin, cargo packaging and transport, organelle fusion, and exocytosis. Understanding these cellular pathways needs advanced cell biology approaches and cell culture models. Although epidermal LBs have well-established functional roles and characterized cargoes, the mechanism of their production is not well understood. Based on the current literature, we propose the following potential models for eLB biogenesis: (a) a sequential maturation of TGN-derived eLB precursor into functional eLBs upon receiving the cargoes from endosomal/lysosomal compartments; (b) the formation of hybrid organelle by fusion of TGN-derived eLB precursors with lysosomes; or (c) the conversion of lysosomes into functional eLBs upon receiving the cargo from Golgi (Figure 4). These models require extensive cellular-level analysis including the cross talk between organelles, followed by *in vivo* verification. Besides, studies aiming to understand the temporal regulation between organelles are essential. The biogenesis of eLB initiates at the spinosum layer and predominates in the granular layer where the turnover of intracellular organelles takes place. Hence, we also propose that studying the sequential modification of intracellular organelles through the epidermal sublayers will possibly decipher the underlying mechanisms of eLB biogenesis and skin homeostasis.

## Author Contributions

SM conceptualized, composed the initial draft of the review, and revised the manuscript. SRGS conceptualized, oversaw the entire project, discussed with co-author, edited the review, and revised the manuscript. Both authors contributed to the article and approved the submitted version.

## Conflict of Interest

The authors declare that the research was conducted in the absence of any commercial or financial relationships that could be construed as a potential conflict of interest.

## Publisher’s Note

All claims expressed in this article are solely those of the authors and do not necessarily represent those of their affiliated organizations, or those of the publisher, the editors and the reviewers. Any product that may be evaluated in this article, or claim that may be made by its manufacturer, is not guaranteed or endorsed by the publisher.
